# Urinary proteomics investigations into contrast-induced acute kidney injury

**DOI:** 10.1371/journal.pone.0258736

**Published:** 2021-10-20

**Authors:** Hong Zhu, Wenwen Chu, Shuai Han, Bihu Gao, Xin Wang

**Affiliations:** 1 Department of Nephrology, Affiliated Zhongshan Hospital of Dalian University, Dalian, China; 2 Department of Nephrology, The First Affiliated Hospital of Wannan Medical College, Wuhu, China; Universita degli Studi di Milano-Bicocca, ITALY

## Abstract

Some patients have a decline in renal function after contrast medium injection, and this phenomenon is called contrast-induced acute kidney injury (CI-AKI); a small number of people even suffer severe renal failure. To date, the mechanism of CI-AKI remains unclear. We aimed to identify novel potential biomarkers in the urine of patients with CI-AKI through LC-MS/MS and bioinformatics analysis. We enrolled patients who underwent coronary angiography (contrast agent: iohexol). The CI-AKI group included 4 cases, and the non-CI-AKI group included 20 cases. We mixed the 4 CI-AKI samples and 20 non-CI-AKI samples. Then, a 0.6 ml urine sample was used for proteome analysis with LC-MS/MS approach. Metascape, ExPASy, and the Human Protein Atlas were utilized for bioinformatics analysis. We obtained 724 and 830 urine proteins from the CI-AKI and non-CI-AKI groups, respectively. The distribution of the pI values and molecular weights (MWs) of postoperative urine proteins showed no significant difference between the CI-AKI group and the non-CI-AKI group. A total of 99differentially expressed proteins (DEPs) were detected, among which 18 proteins were detected only in tubule cells, and 19 proteins were detected in both tubule cells and glomeruli. With GO analysis, the GEPs were mainly associated with immune response and inflammation. Although biomarkers cannot be asserted from this single pilot study, our results may help advance the understanding of the mechanisms of CI-AKI and identify potential novel biomarkers for further investigation.

## Introduction

With the advancement of contrast agent technology, especially the widespread expansion of coronary intervention diagnosis and treatment, many patients undergoing contrast medium injection have a decline in renal function, which is called contrast-induced acute kidney injury(CI-AKI), and a small number of people even suffer severe renal failure. CI-AKI usually refers to the serum creatinine (SCr) level increasing more than 1.5 times from the baseline or the absolute value increasing by more than 26.5 μmol/l (0.3 mg/dl) within two days after the contrast medium injection. In fact, the pathophysiological mechanisms of CI-AKI have not been completely elucidated. Some investigations have proven that the mechanisms of kidney injury include the direct and indirect effects of contrast agents. The nephrotoxic effect of contrast agents on the renal tubular epithelium leads to cell apoptosis, necrosis and ultimately loss of function. The indirect effects of contrast agents may cause a decrease in the levels of vasoactive substances (such as endothelin, nitric oxide, and prostaglandins), resulting in decreased glomerular blood flow and decreased oxygen delivery. In addition, the contrast agent increases blood viscosity, leading to a further decrease in microcirculation blood flow [1].

The true incidence of CI-AKI is currently controversial and different investigations show different incidences, which are mainly related to the patient’s own condition, such as the patient’s basic kidney function, heart function and basic diseases. Thus, the true incidence of CI-AKI remains unclear [2]. In short, there are many obscure aspects of CI-AKI that require further investigation.

At present, traditional SCr has been recognized as a lagging, non-specific marker. Compared with other biological fluids, urine is easy to obtain, can be collected non-invasively, is more abundant and contains a rich source of proteins that are potentially informative of systemic and renal processes. Urine proteomics analysis is an effective and sensitive method that may provide useful information on proteins involved in various pathological processes and the development of diseases [3].

With the development of mass spectrometry (MS) technology, there have been increasing numbers of proteomics investigations into biomarkers of kidney diseases [4–7]; however, research on CI-AKI is rare. In this study, we investigated urine proteins of patients with CI-AKI and non-CI-AKI via liquid chromatography-tandem MS (LC-MS/MS) methods, then performed a comparative analysis of the two groups of urine proteins and determined the differentially expressed proteins (DEPs). After bioinformatics analysis, several proteins were identified that could be novel biomarkers for further research.

## Materials and methods

### Study design

The study protocol was approved by the Health Research Ethics Board, Affiliated Zhongshan Hospital of Dalian University, and all patients provided informed consent (2017–121). We enrolled patients who underwent coronary angiography (contrast agent: iohexol) from Jan.2018 to Dec. 2018 and their individual information was recorded for follow-up. Exclusion criteria included the following: 1.patients with a history of kidney diseases; 2. those suffering from severe heart failure, liver insufficiency or carcinoma; 3. those using potentially nephrotoxic drugs, such as ACE inhibitors (ACEIs), diuretics, non-steroidal anti-inflammatory drugs (NSAIDs), etc., within the last month. Inclusion criteria were patients with estimated glomerular filtration rate (eGFR) of 60 ml/min and age <80 years. The individual in this manuscript has given written informed consent (as outlined in PLOS consent form) to publish these case details. The demographic and clinical data of the patient cohort are described in Table 1. Finally, the CI-AKI group included 4 patients whose SCr increased more than 26.5 μmol/l within 2 days after exposure to contrast medium, and the non-CI-AKI group included 20 patients. We collected 20 ml urine samples 48 h after the procedure. Then, the samples were centrifuged at 3000 ×g for 10 min at 4°C, and the supernatants were stored at −80°C for further analysis.

**Table 1:** Demographic and clinical data.

Group	CI-AKI	non-CK-AKI
Number	4	20
Age(years)	63.75 ± 7.50	64.15 ± 8.09
Sex(male/female)	3/1	7/13
Serum creatinine (μmol/l)	83.75 ± 12.84	66.40 ± 7.51
Blood urea nitrogen (mg/dL)	7.03 ± 0.67	5.82 ± 0.84
Uric acid (μmol/l)	403.75	414.50
eGFR (mL/min/1.72 m^2^)	79.50 ± 7.41	89.79 ± 8.86

Demographic data (mean ± SD); eGFR, estimated glomerular filtration.

#### Urine sample preparation for proteomic analysis

The urine samples were prepared using the FASP method following the procedures described previously with minor modifications [8]. Briefly, we mixed the 4 CI-AKI samples and 20 non-CI-AKI samples. Then, a 0.6 ml urine sample was used for proteome analysis in each group. Disulfide bonds were further reduced by adding 0.01 mol/l DTT and incubating at 95°C for 5 min. Subsequently, iodoacetamide (IAA) was added to reach a final concentration of 20 mM and incubated in darkness for 30 min. Then, solutions were transferred to the Microcon filtration device with a relative molecular mass cut-off of 30,000 (30k filter) was from Sartorius AG (Goettingen, Germany). After centrifugation at 16,000 ×g for 30 min, the precipitates were washed twice with 25 mM Tris-HCl. The proteins were digested overnight by trypsin at a ratio of 1:30 (enzyme/protein, w/w) at 37°C. Finally, the tryptic peptides were collected by centrifugation and washed with water.

#### LC−MS/MS analysis and data processing

All samples were analysed on an EASY-nLC 1000 instrument (Thermo Fisher Scientific, MA, USA) coupled inline to a Q Exactive mass spectrometer (Thermo Fisher Scientific, MA, USA). Peptides were separated on a 15 cm reversed-phase column (150 μm i.d., packed in-house with ReproSil-Pur C18-AQ 3 μm [Dr. Maisch, GmbH, Germany]). The separation elution was 7−23% B for 50 min and 23−40% B for 20 min at a flow rate of 600 nl/min, with buffer A (98% H_2_O, 2% acetonitrile [ACN], 0.1% formic acid [FA]) and buffer B (2% H_2_O, 98% ACN, 0.1% FA). The data were acquired in a data-dependent mode. A full scan was acquired in an Orbitrap from m/z 300 to 1800 at a resolution of 70000. The twenty most intense ions were selected for an MS/MS scan. Peptides were sequentially isolated using a m/z 2.0 isolation window and fragmented by high-energy collisional dissociation (HCD) with a normalized collision energy of 28%. Finally, 5E4 fragment ions were accumulated within a maximum injection time of 60 ms in each MS/MS scan.

Raw files were analysed in the MaxQuant environment (v.1.6.5.0) employing the Andromeda search engine. Protein identification was performed by using a database downloaded from UniProt in August 2019. Enzyme specificity was set to trypsin with up to two missed cleavages. Carbamidomethylation (C) (+57.021 Da) was set as a fixed modification. Oxidation (M) (+15.995 Da) and acetylation (protein N-terminus) (+42.011 Da) were set as variable modifications. The mass tolerances were 10 ppm for the precursor ions and 20 ppm for the fragment ions. High confidence peptide identification was obtained by setting a false discovery rate of <1% at the PSM and protein level with the target-decoy based strategy. At least one unique peptide per protein group was required for identification of proteins and unique and razor peptides were used for quantification of proteins [8]. Matching between runs with a retention time window of 1 min and the label-free quantification (LFQ) algorithm were performed. Then the proteins quantified in triplicates were remained for subsequent analysis, and a two-tailed t test applied with correction for multiple testing (Benjamini-Hochberg) [8]. DEPs were defined as having a p-value <0.05 based on a t-test and a ratio(CI-AKI/non-CI-AKI) >2. The mass spectrometry proteomics data have been deposited to the ProteomeXchange Consortium (http://proteomecentral.proteomexchange.org) via the iProX partner repository [9] with the dataset identifier PXD024271.

#### Bioinformatics analysis

The isoelectric point (pI) values and molecular weight (MW) of the observed proteins were obtained with the “Compute MW/pI” tool from ExPASy (http://web.expasy.org/compute_pi/). We analysed the differential expression of proteins in kidney tissues via the Human Protein Atlas (HPA), which provides abundant information on the tissue and cell distribution of human proteins(www.proteinatlas.org) [10]. Metascape (http://metascape.org) is a powerful gene-list analysis tool for gene annotation and analysis. It can help researchers apply the current popular bioinformatics methods to the analysis of bulk proteins and genes. It has the characteristics of fast updating, inclusion of many databases, being open sourced, and having convenient operation and is accepted by an increasing number of researchers. In our study, Metascape was utilized to perform various enrichment analyses of DEPs. Gene Ontology (GO) terms for biological process(BP), cellular component(CC), and molecular function(MF) categories were included. In addition, Metascape provided information about the expression of genes in human diseases via DisGeNET (http://www.disgenet.org) [11]. As noted on Metascape’s official website, all genes in the genome were used as the enrichment background. Terms with a p-value < 0.01, a minimum count of 3, and an enrichment factor > 1.5 (the enrichment factor is the ratio between the observed counts and the counts expected by chance) were collected and grouped into clusters based on their membership similarities [12].

## Results

### 1. Distribution of pI and MW of observed urine proteins

**Fig 1:**
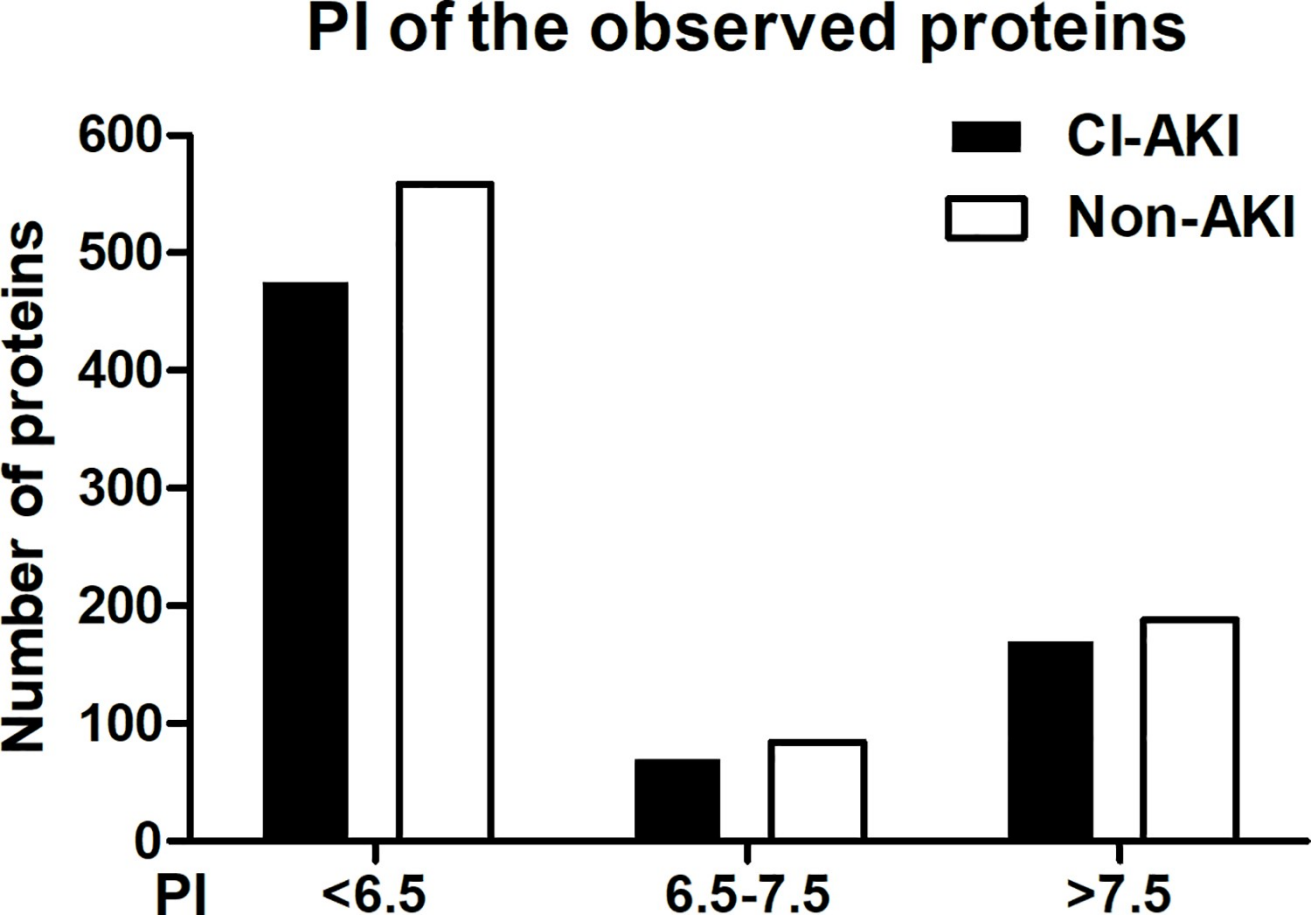
pI of the observed proteins. Distribution of pI values of the observed urine proteins from the CI-AKI and non-CI-AKI groups. No significant difference was found.

**Fig 2:**
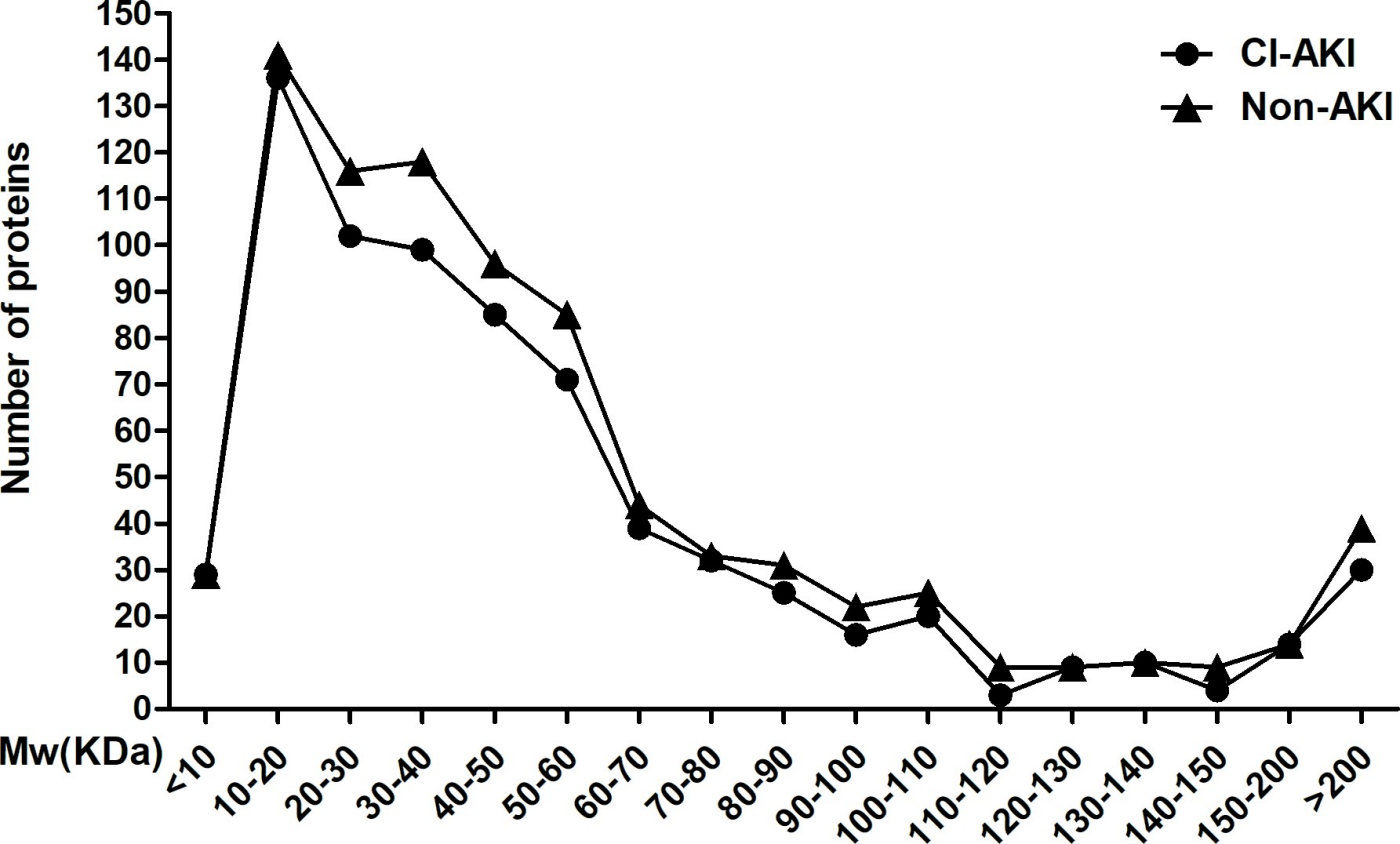
MWs of the observed proteins. Distribution of MWs of the observed urine proteins from the CI-AKI and non-CI-AKI groups. No significant difference was found.

We obtained 724 and 830 pI values of the postoperative urine proteins from the CI-AKI and non-CI-AKI groups, respectively. Then, we divided them into three groups based on pI<6.5, 6.5 to 7.5 and >7.5, and the distribution of urine proteins was observed. We found that most proteins had a pI value<6.5, some has a pI>7.5, and the least number of proteins had a pI of 6.5–7.5 (Fig 1). We also observed the MW of the urine proteins. As shown in Fig 2, most proteins had MW<70 kDa, and the proteins with MW ranging from 10 kDa to 20 kDa accounted for the largest portion. Neither the pI nor MW of postoperative urine proteins showed a significant difference between the CI-AKI group and the AKI group.

### 2. Expression in the kidney

**Fig 3:**
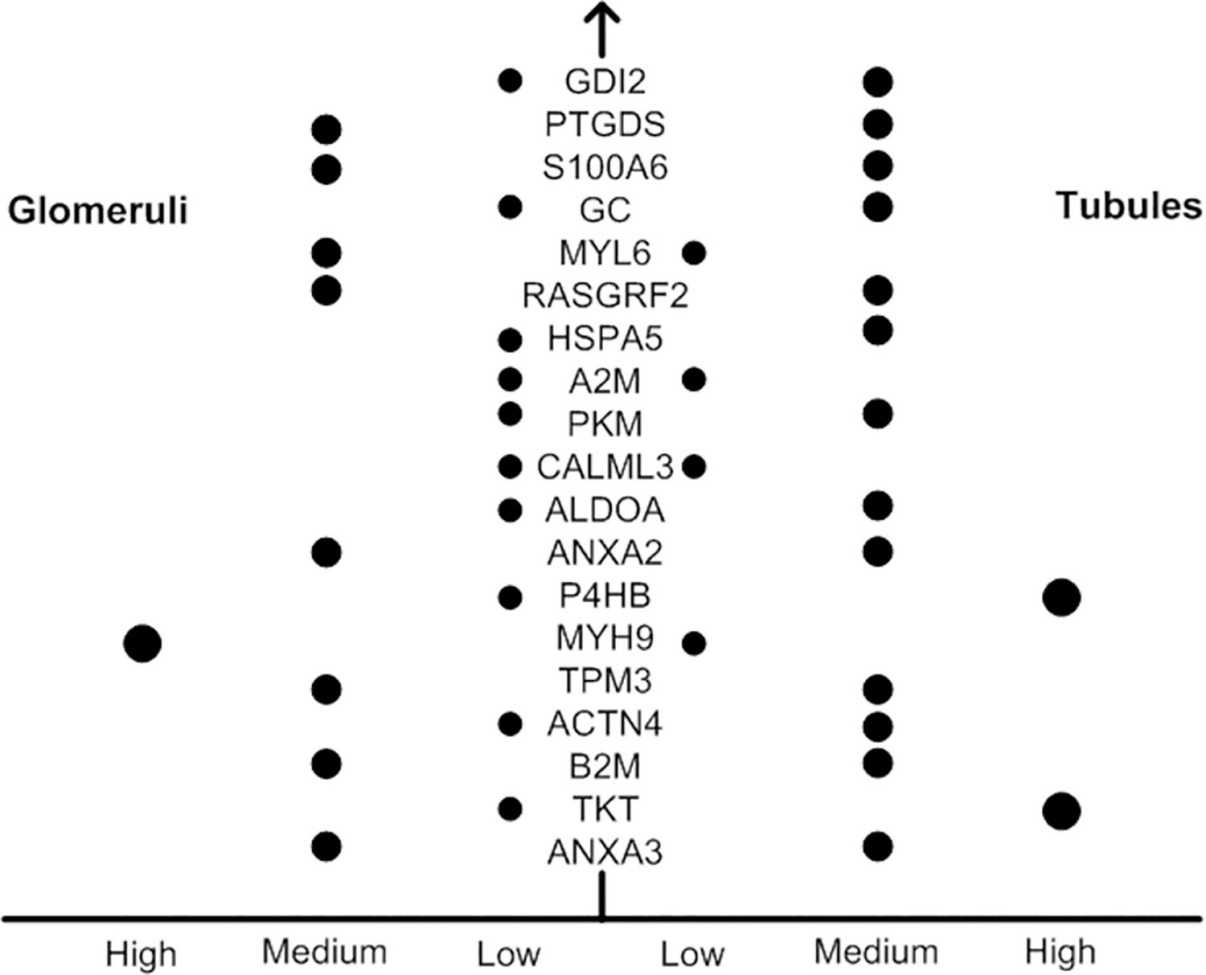
Protein expression in both glomeruli and tubules. Differential expression of proteins in both glomeruli and tubules, with the units low, medium and high annotated by the HPA.

**Fig 4:**
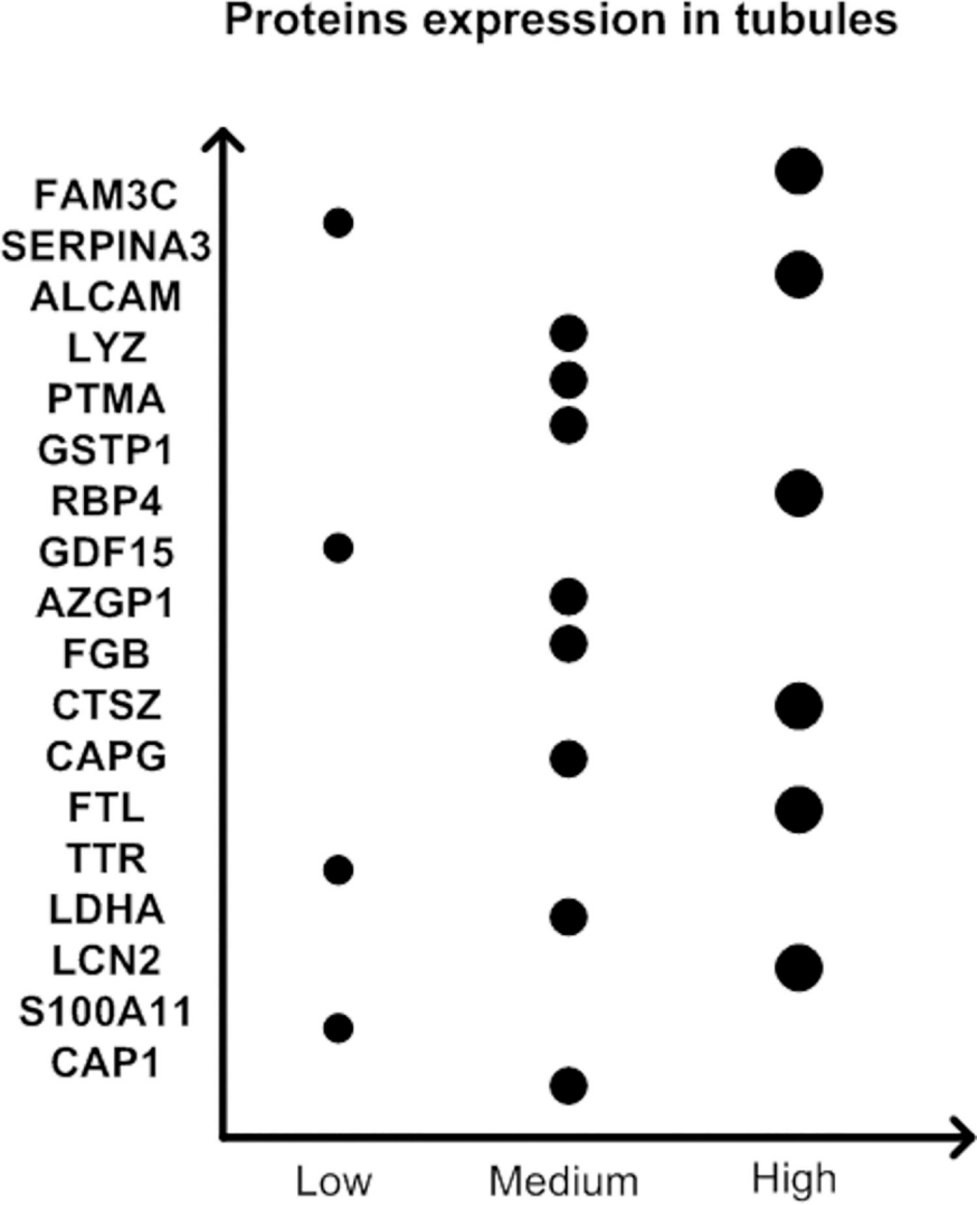
Protein expression in both tubules. Differential expression of proteins in only tubule cells, with the units low, medium and high.

As shown in the S1 Table, a total of 99DEPs were detected, among which 39 proteins were shown in the kidney, as depicted in Figs 3 and 4. Eighteen and 2 proteins that were shown to originate only from the tubules and glomeruli, respectively, and 19 proteins were shown to originate from both the tubules and glomeruli.

### 3. GO analysis

**Fig 5:**
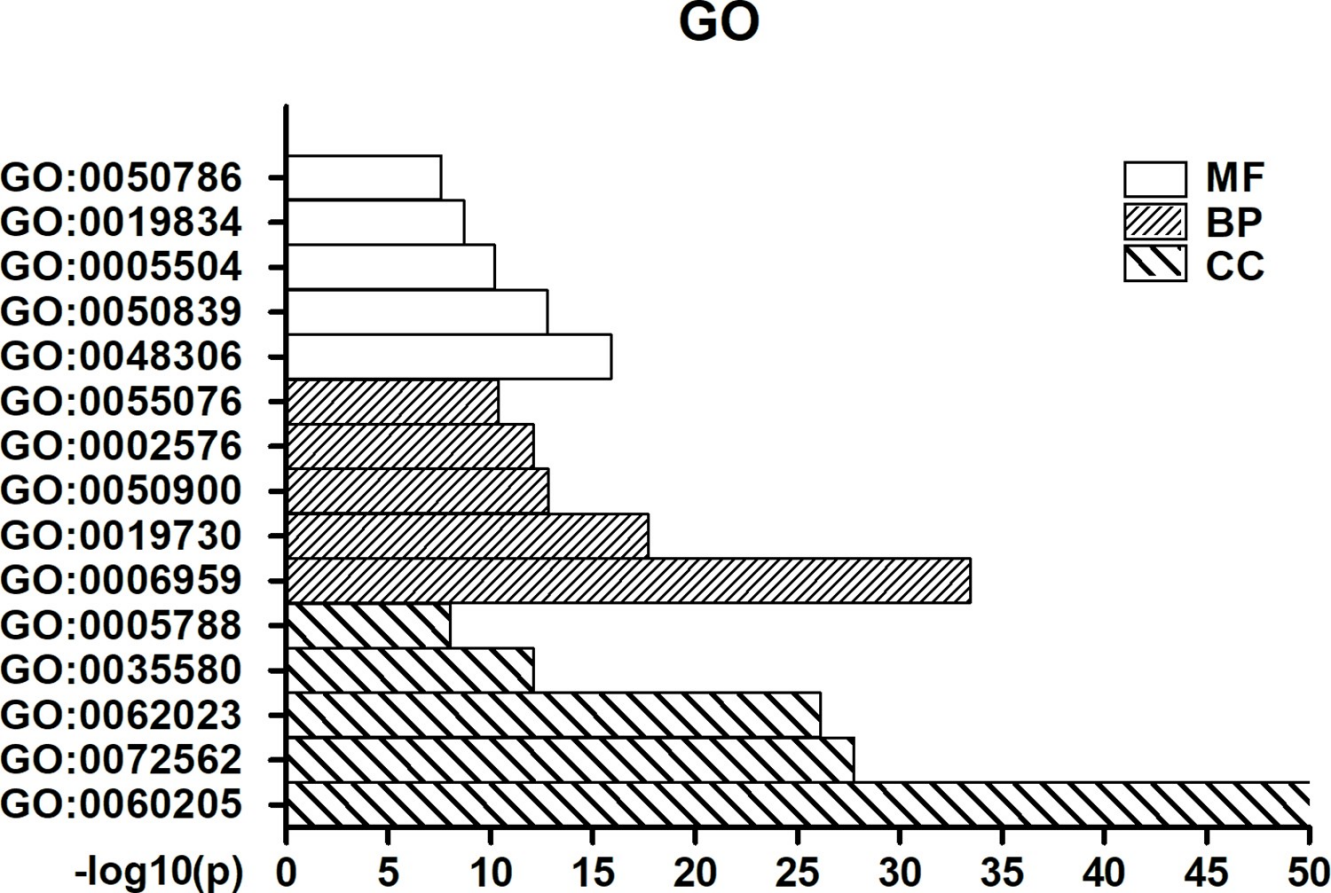
GO analysis. MF: molecular function; BP: biological process; CC: cellular component; GO: 0050786 RAGE receptor binding; GO: 0019834 phospholipase A2 inhibitor activity; GO: 0005504 fatty acid binding; GO: 0050839 cell adhesion molecule binding; GO: 0048306 calcium-dependent protein binding; GO: 0055076 transition metal ion homeostasis; GO: 0002576 platelet degranulation G; GO: 0050900 leukocyte migration; GO: 0019730 antimicrobial humoral response; GO: 0006959 humoral immune response; GO: 0005788 endoplasmic reticulum lumen; GO: 0035580 specific granule lumen; GO: 0062023 collagen-containing extracellular matrix; GO: 0072562 blood microparticle; GO: 0060205 cytoplasmic vesicle lumen.

As shown in Fig 5, GO analysis demonstrated that most proteins were assigned to functions involved in BPs, including humoral immune response, antimicrobial humoral response, leukocyte migration, platelet degranulation, and transition metal ion homeostasis. The CC associations included cytoplasmic vesicle lumen, blood microparticle, collagen-containing extracellular matrix, specific granule lumen, and endoplasmic reticulum lumen. The MFs included calcium-dependent protein binding, cell adhesion molecule binding, fatty acid binding, phospholipase A2 inhibitor activity and RAGE receptor binding.

### 4. Association with human diseases

**Fig 6:**
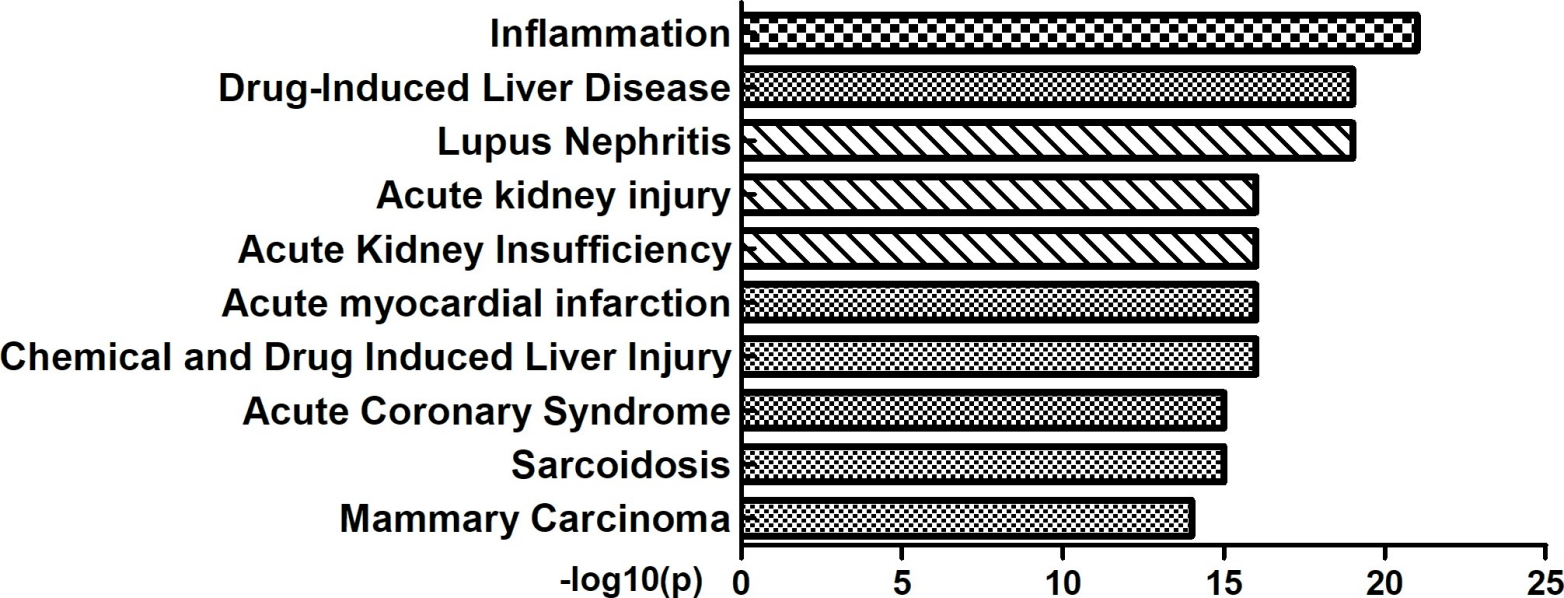
DEPs related to human diseases. Differential expression of proteins are related to many human diseases, including inflammation, lupus nephritis, acute kidney injury and acute kidney insufficiency.

We utilized Metascape to further investigate the relations between detected proteins and diseases. As shown in Fig 6, the relevant diseases included inflammation, drug-induced liver disease, lupus nephritis, acute kidney injury, acute kidney insufficiency, acute myocardial infarction, chemical- and drug-induced liver injury, acute coronary syndrome, sarcoidosis and mammary carcinoma.

## Discussions

The incidence of CI-AKI varies from 5% to 20% among hospitalized patients [13, 14]. However, research on the pathophysiological mechanism and biomarkers of CI-AKI has not been profound so far, especially research with LC-MS/MS methods. Therefore, we identified the urine proteins of CI-AKI patients with LC-MS/MS methods and compared the differences in urine protein expression between AKI patients and non-CI-AKI patients with bioinformatics analysis, providing a basis for further research on CI-AKI.

In our study, we first detected the urine proteins of CI-AKI patients and non-CI-AKI patients and then analysed the distribution of urine proteins based on their pI and MW. The results showed that the 10 kDa-20 kDa proteins in urine accounted for the largest portion, and with increased MW, the number of proteins gradually decreased; however, no significant difference was found between the CI-AKI and non-CI-AKI samples. It is known that when the glomerulus is severely damaged, a large number of negatively charged and large MW proteins appear in the urine. Nevertheless, we did not find this phenomenon in our study, which indicated that the glomerulus might not be severely damaged in patients with CI-AKI. In the following analysis, we found that DEP expression in kidney tissues was mainly located in tubules. Although some proteins were expressed in both glomeruli and tubules, the level of those expressed in glomeruli was lower than that of proteins expressed in tubules. This result suggested that tubules may play a more important role in CI-AKI. To date, many studies have identified several biomarkers capable of the detection of kidney injury including NGAL, KIM-1, IL-18 and some other important molecules. In our study, several DEPs were proved to have associations with kidney injury. However, the roles of most of the DEPs in CI-AKI are still unclear and need more investigations.

Young *et al*. reported that urine NGAL (LCN2) and S100-P protein levels increased significantly which are promising biomarkers for prediction of AKI in preterm infants [15]. Similarly, we found that NGAL and S100-P protein were up-regulated in CI-AKI patients with ratios of 4.25 and 6.63, respectively (S1 Table). Urine NGAL is postulated to be a highly sensitive marker of AKI, specifically of tubular cell damage rather than a decrease in glomerular filtration [15]. Our study showed that urine NGAL levels increased in CI-AKI patients, which implied that NGAL is possible a valuable biomarker for prediction of CI-AKI. The S100 proteins are the largest subgroup within the superfamily of EF-hand Ca2+ -binding proteins. The expression of S100 proteins has been investigated in several malignant neoplasms, especially renal neoplasms [16]. Young *et al*. identified that S100P could be a novel biomarker for AKI in their study [15]. Similarly, we found that urinary S100P levels significantly increased in patients with CI-AKI which implied that S100P might play an important role in the occurrence of CI-AKI.

Annexins are Ca2^+^ and phospholipid-binding proteins, some of which have been considered to participate in the regulation of membrane organization and trafficking, as well as the regulation of ion currents across membranes [17, 18]. As ischemic renal dysfunction significantly contributes to apoptosis, annexin A5 can be a biomarker for predicting AKI [15]. Urine annexin A5 did not show significant differences in the patients with CI-AKI in our study; however, some other proteins of the annexin family increased significantly including annexin A1, annexin A2 and annexin A3. ANXA2 is mainly located in the cytoplasm and translocates to the cell membrane. It is one of the calcium-dependent phospholipid-binding proteins [19]. It has many functions, including exocytosis, cell-matrix interactions, cell motility, endocytosis, signal transduction, transcription, mRNA transport, and DNA replication [20]. A recent study suggested that nodular glomerulosclerosis partially results from DNA damage in the glomerulus, which induces collagen type VI secretion from human renal glomerular endothelial cells via ataxia telangiectasia and Rad3-related and ANXA2-mediated pathways [21]. Consistent with our results, ANXA2 also showed elevated expression in injured human proximal tubular epithelial cells stimulated by calcium oxalate monohydrate [22]. Therefore, we think that ANXA2 would probably be a valuable biomarker for investigation in tubulointerstitial disease.

Prasad reported several most commonly investigated up-regulated proteins in AKI determined by proteomic methods including NGAL, albumin, β2-microglobulin and α-1-antitrypsin [23]. In our study, β2-microglobulin (B2M) andα-1-antitrypsin (SERPINA1) were up-regulated in CI-AKI patients with ratios of 7.4 and 4.27, respectively (S1 Table). β2M is an 11.8 kDa protein that is used to evaluate the function of tubules because it is filtered by glomeruli and reabsorbed by renal proximal tubules [24]. A study confirmed that baseline β2M was an independent predictor for CI-AKI [25].

In addition, we found that several DEPs such as RBP4 and GDF15are related to various renal diseases. Retinol-binding protein is a small MW plasma protein that is completely absorbed by the renal tubules after being filtered from the glomerulus under normal conditions. Its increased concentration in the urine reflects impaired renal tubular reabsorption [26]. The molecular mass of RBP4 is 21 kDa, and RBP4 is mainly secreted by the liver and degraded by the kidney [27]. It has been reported that the serum level of RBP4 in individuals with diabetes is significantly higher than that in healthy individuals; moreover, it increases with a decline in renal function [28, 29]. GDF15, also known as macrophage inhibitory cytokine(MIC-1), is a regulator of the inflammatory response, dendritic cell maturation and peripheral blood mononuclear cell proliferation [30, 31]. It is a member of the transforming growth factor-β(TGF-β) cytokine family and an inflammation-associated protein. It has been reported that GDF15 plays an important role in some metabolic diseases [32, 33]. Recently, a study showed that the induction of glomerulonephritis in mice can induce systemic GDF15 expression. Moreover, GDF15-deficient mice showed increased levels of the CXCR3 receptor in activated T cells and increased levels of proteinuria with aggravated crescent formation and mesangial expansion in anti-GBM nephritis. This study suggested that CXCL10/CXCR3-dependent signalling promotes T cell infiltration into the organ during GDF15-regulated acute inflammatory processes [34]. Thus, GDF15 has important value for further investigation.

Through GO analysis, we found that DEP enrichment of BPs mainly included humoral immune response, antimicrobial humoral response, leukocyte migration, platelet degranulation, and transition metal ion homeostasis, which are closely related to the immune system and inflammatory response. This result indicated that the occurrence of CI-AKI was probably associated with immunity and inflammation. In the following analysis, we found that DEPs are related to many human diseases, including inflammation, lupus nephritis, acute kidney injury, and acute kidney insufficiency. This finding suggested that the relationships between DEPs and kidney disease are reliable and that inflammation may participate in CI-AKI progression. Recently, statins have been shown to reduce the risk of contrast-induced nephropathy (CIN) by reducing inflammatory and immunomodulatory processes [35, 36]. Reactive oxygen species (ROS), such as superoxide, hydrogen peroxide and hydroxyl radicals, are actively involved in inflammatory responses that are generated during renal parenchymal hypoxia induced by contrast medium [37]. Consistent with our bioinformatics analysis, these studies suggested that inflammation and immunity are very important factors for CI-AKI.

In our study, the significance of most of the identified proteins in human disease is still uncertain and needs additional investigation. There are some limitations in our study. First, this study lacks tissue-related evidence because most patients with CI-AKI cannot undergo renal biopsy. Second, the sample size in the study was small; thus, additional multi-centre clinical investigations will be performed to validate these significant DEPs in the future.

## Conclusions

Proteomics technology is efficient and sensitive and can discover novel biomarkers effectively. Although biomarkers cannot be asserted from this single pilot study, our results may help advance the understanding of the mechanisms of CI-AKI and identify potential novel biomarkers for further investigation.

## Supporting information

S1 Table Protein information. List of detected proteins.
(XLSX)
